# Imaging and visualizing SARS-CoV-2 in a new era for structural biology

**DOI:** 10.1098/rsfs.2021.0019

**Published:** 2021-10-12

**Authors:** Kendra E. Leigh, Yorgo Modis

**Affiliations:** ^1^ Molecular Immunity Unit, Department of Medicine, MRC Laboratory of Molecular Biology, University of Cambridge, Francis Crick Avenue, Cambridge CB2 0QH, UK; ^2^ Cambridge Institute of Therapeutic Immunology and Infectious Disease (CITIID), University of Cambridge School of Clinical Medicine, Cambridge CB2 0AW, UK

**Keywords:** SARS-CoV-2, structural biology, cryo-electron tomography, X-ray crystallography, cryo-electron microscopy, multiscale imaging

## Abstract

The SARS-CoV-2 pandemic has had a global impact and has put scientific endeavour in the spotlight, perhaps more than any previous viral outbreak. Fortuitously, the pandemic came at a time when decades of research in multiple scientific fields could be rapidly brought to bear, and a new generation of vaccine platforms was on the cusp of clinical maturity. SARS-CoV-2 also emerged at the inflection point of a technological revolution in macromolecular imaging by cryo-electron microscopy, fuelled by a confluence of major technological advances in sample preparation, optics, detectors and image processing software, that complemented pre-existing techniques. Together, these advances enabled us to visualize SARS-CoV-2 and its components more rapidly, in greater detail, and in a wider variety of biologically relevant contexts than would have been possible even a few years earlier. The resulting ultrastructural information on SARS-CoV-2 and how it interacts with the host cell has played a critical role in the much-needed accelerated development of COVID-19 vaccines and therapeutics. Here, we review key imaging modalities used to visualize SARS-CoV-2 and present select example data, which have provided us with an exceptionally detailed picture of this virus.

## 1. Background

COVID-19 grew to pandemic scale with alarming speed and has continued to claim a heavy toll. However, the advent of the pandemic in late 2019 was fortuitous in more ways than one. A new generation of vaccine platforms was on the cusp of clinical maturity, making it possible for multiple highly effective vaccines to be developed and approved within a year of the outbreak. Perhaps just as importantly, SARS-CoV-2 emerged at the inflection point of a technological revolution in macromolecular imaging by cryo-electron microscopy (cryo-EM), fuelled by a confluence of major advances in sample preparation, optics, detectors and image processing software for electron microscopy [1–3]. Together, these advances enabled us to visualize the molecular structure of SARS-CoV-2 and its components more rapidly, in greater detail, and in a wider variety of biologically relevant contexts than would have been possible even a few years earlier. The resulting wealth of ultrastructural information on SARS-CoV-2 and how it interacts with the host cell played a critical role in the remarkable and much-needed acceleration in the development of COVID-19 vaccines and therapeutics. Here, we review the key imaging modalities used to visualize SARS-CoV-2 and present select example data that have provided us with an exceptionally detailed picture of SARS-CoV-2 on a wide range of scales at every stage of the viral life cycle.

## 2. The resolution spectrum

**Figure 1.**
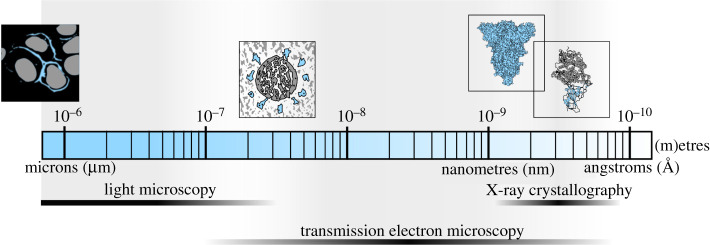
Recent technological advances have allowed us to image SARS-CoV-2 and its components on a continuous resolution spectrum up to the near-atomic scale. From left to right: a schematic of spike protein on the surface of cells as it would appear if visualized by fluorescent light microscopy; a schematic of a SARS-CoV-2 virion as it would appear if visualized by cryo-electron tomography; a cryo-EM reconstruction of the spike protein trimer (EMD-21374); a model based on the crystal structure of ACE2 (angiotensin-converting enzyme 2) in complex with the receptor binding domain (RBD) of spike protein (PDB 6M0J).

**Figure 2.**
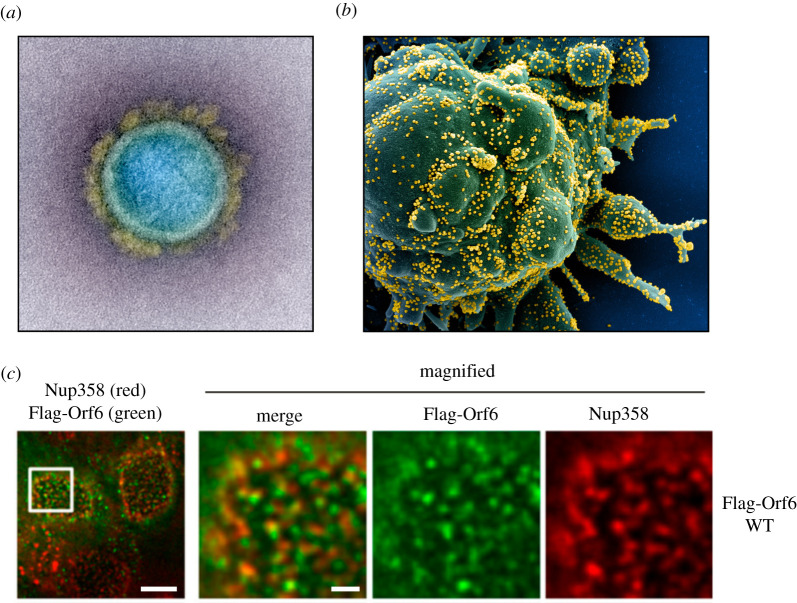
SARS-CoV-2 and the cell. (*a*) Electron microscopy plays an important confirmatory role in diagnosis and determining subcellular localization. A false-colour transmission electron micrograph of SARS-CoV-2 isolated from a patient. Coronaviruses are approximately 80–120 nm in diameter [7]. (*b*) A false-colour scanning electron micrograph of an apoptotic cell (green) infected with SARS-CoV-2 isolated from a patient. The virions can be observed as small yellow spheres. (*a*) and (*b*) are adapted from images of ‘Novel Coronavirus SARS-CoV-2’ published by NIAID and licensed under CC BY 2.0 [8,9]. To view a copy of this license, visit https://creativecommons.org/licenses/by/2.0/. (*c*) Fluorescence microscopy can show subcellular localization. Co-localization of FLAG-tagged SARS-CoV-2 protein Orf6 with nuclear pore complex protein Nup358 in HEK293T cells as imaged by stimulated emission depletion (STED) super-resolution microscopy. Scale bar in the left-most panel is 5 µm. Scale bar in the magnified panels is 1 µm. This panel is adapted from ref. [10] and licensed under CC BY 4.0. To view a copy of this license, visit https://creativecommons.org/licenses/by/4.0/.

Microscopes have been used to image viruses since viruses were discovered in the late nineteenth century [4], but only in the last decade or so have technological advances in electron microscopy allowed us to image viruses on a continuous resolution spectrum. With dimensions smaller than the wavelength of visible light, viruses could not be visualized directly until the invention of the electron microscope in the 1930s [5,6] (figure 1). Since then, electron microscopy has been the method of choice for identifying viruses, based on their size, shape, ultrastructural features and tissue distribution. Our first glimpses of SARS-CoV-2 were in electron micrographs taken within weeks of the outbreak (figure 2) [11].

The images derived from electron microscopy are often noisy, making the features contained therein appear fuzzy and indistinct. This effect can be compounded by the treatments that samples must be subjected to prior to imaging in a conventional transmission electron microscope. Chemical fixation, use of contrast agents (e.g. uranyl acetate), drying and embedding of the sample in plastic resin limit the imaging resolution and distort the sample, yielding results that fall short of the molecular detail needed to be useful in vaccine and antiviral drug design. X-ray crystallography of viral proteins, macromolecular assemblies and occasionally entire viruses can provide the necessary resolution but requires the object of interest to be crystallized. In practice, this limits crystallographic structure determination to homogeneous, highly soluble and relatively small (rarely larger than 200 kDa) macromolecules. The irregular, non-spherical shape of SARS-CoV-2 virions, therefore, precludes crystallization.

Up until the mid-2010s, in most cases, the limitations of these techniques left a large gap in achievable imaging resolutions between conventional transmission electron microscopy (with resolutions on the order of nanometres) and X-ray crystallography (with resolutions on the order of angstroms). For the most part, determining the structure of an entire virion, alone or in the context of a host cell, remained inaccessible to high resolution structure determination by any method. This began to change with the Nobel-prize-winning development by Dubochet and colleagues [12] of cryogenic vitrification of aqueous samples, including viruses, for cryo-EM imaging. These early cryo-EM micrographs were essentially free of preservation artefacts but still suffered from blurring and poor signal-to-noise ratios. Additional developments, most notably direct electron detectors, maximum likelihood image classification and averaging algorithms, and improved tomographic reconstruction and subtomogram averaging (STA) algorithms, which became widely available from 2013 onwards, really brought the field to the fore [1–3]. These advances ushered in a new era in structural biology in which the structures of large asymmetric macromolecular assemblies can now be reconstructed at a resolution sufficient to build atomic models (between 0.1 nm and 1 nm resolution). While the use of contrast reagents and conventional electron microscopy on chemically fixed samples continues to be the method of choice for rapid identification of viruses, particularly, within infected cells or tissues, cryo-EM extends our ability to obtain high-resolution information of entire viruses and macromolecular assemblies that cannot be crystallized. When SARS-CoV-2 emerged, these tools had matured, allowing them to be rapidly applied to the virus and its components on a continuous resolution spectrum, through a combination of conventional electron microscopy, cryo-EM and X-ray crystallography (figure 1). The snapshots from these structural studies have been provided a more dynamic biological context through the use of light microscopy, which can be used to track and identify viruses in real-time as they move through the host cell (figure 2).

## 3. Finding and tracking SARS-CoV-2 in the cell

Transmission electron microscopy (TEM) of tissue or fluid samples has historically played a key role in the identification of new viruses. SARS-CoV-2 was first imaged by TEM within weeks of the initial outbreak (figure 2*a*) [11]. Micrographs provided important visual confirmation of sequencing data that the new virus, displaying the prototypic ‘corona’ of glycoproteins, was a coronavirus. TEM also supported biochemical data suggesting that virus entry can occur at the plasma membrane or via endosomes [13–15]. Scanning electron microscopy (SEM) of infected cells showed large numbers of virus particles released from a single cell indicating that infection was acute and highly productive (figure 2*b*). However, these TEM and SEM micrographs alone cannot provide the molecular detail needed to understand the specific protein–protein interactions at the heart of the biological processes essential to the virus life cycle; for example, how SARS-CoV-2 recognizes host cells or how antibodies neutralize the virus. Moreover, these images are static, yielding no clues about the timescales of virus-cell entry, establishment of replication sites—‘virus factories’, and emergence of newly replicated virions from the cell.

Electron microscopy samples must be imaged in a vacuum to prevent scattering of the electron beam, thus making it impossible to study the dynamics of biological processes in living specimens. By contrast, light microscopy can be performed on live cells, allowing for example, tracking of fluorescently labelled viruses in real-time as they travel through the cell. Despite its lower imaging resolutions, light microscopy powerfully complements electron microscopy by providing a time dimension. Moreover, the use of genetically or chemically encoded fluorescent labels allows targets of interest, such as viral proteins, to be identified among the tens of thousands of other proteins that make up the cell (figure 2*c*). Fluorescence microscopy of virus-infected cells has been used to identify possible cell entry pathways for SARS-CoV-2, the architecture and subcellular location of virus replication sites, the effects of infection on cell morphology and the timings of each of these steps [16,17]. For example, fluorescence microscopy revealed that lentiviruses pseudotyped with SARS-CoV-2 spike glycoprotein can take advantage of clathrin-mediated endocytosis pathways, where ubiquitous cathepsins take the place of TMPRSS2 (transmembrane protease serine 2) and prime the spike glycoprotein for entry within endosomes [13]. Fluorescence microscopy imaging had also shed light on other biological properties such as the membrane fusion activity of the spike glycoprotein, subcellular localization of viral proteins and inhibitor activity [10,18,19].

Complementary light and electron microscopy modalities, such as cryo-CLEM (cryo-correlative light and electron microscopy), have been applied to multiple viruses to gain an understanding of virus life cycles at the lower end of the resolution spectrum. Application of these modalities to SARS-CoV-2 allowed rapid identification, tracking of infection within the cell, including co-localization with cellular components, and ultrastructural characterization of larger virus-associated assemblies (such as replication sites). This type of information is necessary, not only to provide biological context but also to gain an understanding that can be leveraged in the development of antiviral approaches.

## 4. The cryo-EM revolution—impact on SARS-CoV-2 imaging

What sets the imaging and visualization of SARS-CoV-2 apart from work done on previous epidemics is the astonishing amount of high-resolution structural information that was gleaned and the pace at which it was delivered. This was made possible largely by transformative technological advances in cryo-EM (reviewed in [1–3]), and by an unprecedented mobilization of the scientific enterprise towards COVID-19-related research across public and private sectors. The majority of the cryo-EM-derived structural information on SARS-CoV-2 has come from image reconstructions determined by single-particle averaging (SPA). In SPA, thousands, or even millions, of images of a protein or macromolecular assembly in different orientations, but ideally in identical conformations, are extracted from cryo-EM micrographs, classified, aligned and then averaged to calculate a three-dimensional structure. The power of this averaging process greatly reduces the high background noise present in cryo-EM images, which have low signal to noise ratios due to the low scattering potential and therefore low inherent contrast of biological material, compounded by sample movement and radiation damage caused by the microscope's electron beam.

Cryo-EM has existed for decades as a structural technique, but more recent advances have taken the field to a state where near-atomic resolutions are readily achievable. Maximum likelihood statistical algorithms, as implemented for example in the RELION software package [20], greatly improve the alignment accuracy, and hence the effective resolution, of SPA image reconstruction. However, this process is computationally expensive. Computation and data storage requirements that would have been prohibitive even 10 years ago have recently become tractable through more affordable data storage, GPU (graphics processing unit)-based parallelization and the development of faster CPUs (central processing units). In parallel, the introduction of direct electron detectors, based on the principles behind the active-pixel CMOS (complementary metal-oxide-semiconductor) sensors used in consumer digital cameras, has significantly reduced detector noise and allowed for the acquisition of movies rather than still images [21]. With data acquired as a set of movies, some of the obstacles to high-resolution reconstructions, such as blurring, can be overcome with computational approaches including motion-correction algorithms [22]. Together these advances, along with improved sample preparation workflows and hardware, have dramatically improved the capability of cryo-EM image reconstruction by SPA. As a result, resolutions comparable to those achieved in X-ray crystallography can now routinely be achieved by cryo-EM. The key advantage of cryo-EM over crystallography, however, is that cryo-EM can be used to determine structures of a wide variety of macromolecules that cannot be readily crystallized because they are too large, too flexible, insufficiently soluble (for example because they contain transmembrane domains or lipid anchors) or simply because they are not available in sufficient quantity or purity.

### 4.1. The SARS-CoV-2 spike structure

**Figure 3.**
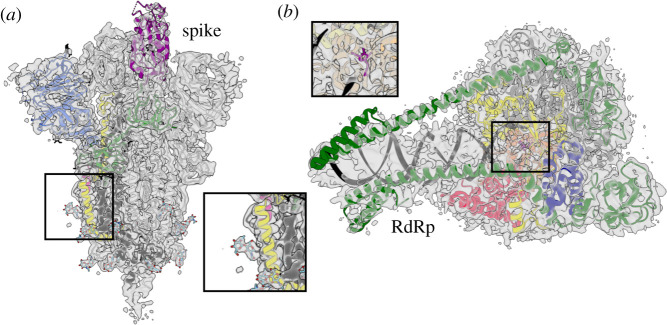
A technological revolution in cryo-EM has opened new imaging frontiers. (*a*) Cryo-EM map of stabilized trimeric spike ectodomain (EMD-21375) fitted with its corresponding atomic model (PDB 6VSB). Inset shows a closer view of the correspondence between the map and the model. N-terminal domain, blue: RBD, purple, C-terminal domain, green; fusion peptide, red; HR1, yellow. (*b*) Cryo-EM map of the apo RNA-dependent RNA polymerase (EMD-11007) fitted with its corresponding atomic model (PDB 6YYT). Remdesivir is shown in purple, positioned at the end of the RNA product. Its position is based on the alignment of a remdesivir-bound structure (PDB 7BV2) with the apo model. A clipped perspective of the remdesivir binding site is highlighted in the inset. nsp7, blue; nsp8, green; polymerase domains: ‘fingers’, yellow; ‘palm’, orange; ‘thumb’, red.

The cumulative technological advances in multiple fields of structure determination mean that within little over a year of the outbreak, structures have been determined of nearly all of the constituent proteins in SARS-CoV-2 at resolutions sufficient to allow atomic models to be built [23]. Arguably, the best example of the speed at which structural biology, and cryo-EM in particular, can now operate is illustrated by structures of the spike (S) glycoprotein of SARS-CoV-2. In March 2020, only a few months after the sequencing of the new virus, McLellan and colleagues [24] reported a cryo-EM structure of the soluble ectodomain of S in a stabilized trimeric prefusion conformation, determined by SPA to 3.5-Å resolution (figure 3*a*). Additional cryo-EM structures of the spike trimer quickly followed (for example, [25–29]), including those in complex with neutralizing antibody fragments [30] or other binding partners, at a pace that would not have been possible using crystallography or other previously available approaches. To date, there are well over 100 cryo-EM structures of the spike or its subcomponents [31], and the highest resolution structure currently available was determined to a resolution of 2.4 Å, which is at least on par with the average resolution of crystal structures of similarly sized molecules [32].

S is the single most important antigen on the virion surface and is the primary target of most neutralizing antibodies against SARS-CoV-2 [33]. Consequently, S was the expressed antigen selected for the first wave of SARS-CoV-2 vaccines, including those from Pfizer/BioNTech, Moderna and Oxford–AstraZeneca. Several of the S protein constructs used for cryo-EM structure determination contain various stabilizing mutations, most notably proline substitutions at residues 986 and 987, which also help preserve S in the pre-fusion conformation [24,26,29,32,34]. Previous structural and immunological studies on spike proteins from other coronaviruses have shown that such stabilizing mutations can result in improved expression stability and immunogenicity [35]. This previous work was fortuitous because knowledge of these mutations could be directly and rapidly applied to SARS-CoV-2 vaccine formulations [29,36], highlighting how in this case structural biology and vaccinology mutually benefitted each other. The spike structures have also served as excellent platforms for the analysis and prediction of the effects of any point mutations acquired by new isolates or variants of the virus [37,38]. Furthermore, the rapid turnaround in cryo-EM structure determination means that spike structures can continue to be determined to address specific contexts, for example with antibodies or inhibitors bound, and thus continue to inform treatment and prevention strategies.

### 4.2. The RNA polymerase structure

SARS-CoV-2 relies on an RNA-dependent RNA polymerase (RdRp) to replicate and transcribe its positive-stranded RNA genome. Viral RNA polymerases have historically proven to be attractive drug targets. Various small molecules have been identified that inhibit specific viral RNA polymerases without affecting our essential cellular RNA polymerases. AZT (3′-azido-3′-deoxythymidine) which inhibits the reverse transcriptase of HIV (a type of RNA polymerase), paved the way for the successful clinical suppression of chronic HIV infection we have achieved today [39]. The cure for hepatitis C virus (HCV) developed by Gilead Sciences, one of the pharmaceutical industry's most notable recent successes in the treatment of infectious diseases, includes the use of an HCV RdRp inhibitor [40,41].

The SARS-CoV-2 RdRp was, therefore, immediately identified as one of the most important targets for structure-based drug discovery. With a molecular mass under 150 kDa, the SARS-CoV-2 RdRp would until recently have been too small to image by cryo-EM. With current technology, however, two groups were able to independently determine cryo-EM structures of the SARS-CoV-2 RdRp with an RNA template-product complex bound at 2.9 Å resolution by early 2020 (figure 3*b*) [42,43]. The structure revealed the active site architecture and served as the ideal platform to accelerate the search for small molecule inhibitors of RdRp activity. Remdesivir, originally developed by Gilead as a potential treatment for RNA viruses and used in clinical trials against Ebola virus [44], became the first treatment for COVID-19 requiring hospitalization to be approved by the U.S. Food and Drug Administration in October 2020 [45,46]. Shortly afterwards, cryo-EM structures of SARS-CoV-2 RdRp with remdesivir bound in the active site showed how the incorporation of remdesivir by the RdRp into nascent RNA products blocks translocation of the transcript and thereby stalling transcription (figure 3*b*) [47,48]. This example illustrates how the availability of high-resolution target structures allows not only structure-based drug design but also elucidation of drug mechanisms, which in turn allows for future improvement of specificity and function.

### 4.3. Cryo-electron tomography of intact virions

Although cryo-EM structures of soluble purified recombinant spike proteins were rapidly determined by SPA, visualizing the structure and distribution of S trimer in the context of intact virions required a different approach. Cryo-EM structure determination by SPA relies on averaging two-dimensional images of many highly similar copies of a macromolecule. But larger biological assemblies tend to be more structurally heterogeneous, due to the intrinsic flexibility of their protein, lipid or nucleic acid constituents. For example, no two mitochondria, bacterial flagella or nuclear pores are exactly identical. Similarly, many viruses, including SARS-CoV-2, have intrinsically heterogeneous structures. Because each SARS-CoV-2 virion has a slightly different shape and spike distribution, structure determination by SPA is not feasible. However, asymmetric assemblies can be visualized by another EM-based approach: tomography. Tomographic reconstruction of cryo-EM micrographs taken of the same region of interest with the sample tilted through a series of angles results in a three-dimensional volume (tomogram) that can then be analysed. Recent advances in image processing algorithms for cryo-electron tomography (cryo-ET) have improved image alignment accuracy and allowed for averaging of repeating objects within a volume reconstruction, a process analogous to SPA known as STA [49,50]. These advances have allowed for STA reconstructions of macromolecules found within large asymmetric assemblies to be determined to remarkably high resolutions in some cases (3.4 Å for an HIV capsid structure, for example [51,52]), although subnanometre resolutions are still not routine.

**Figure 4.**
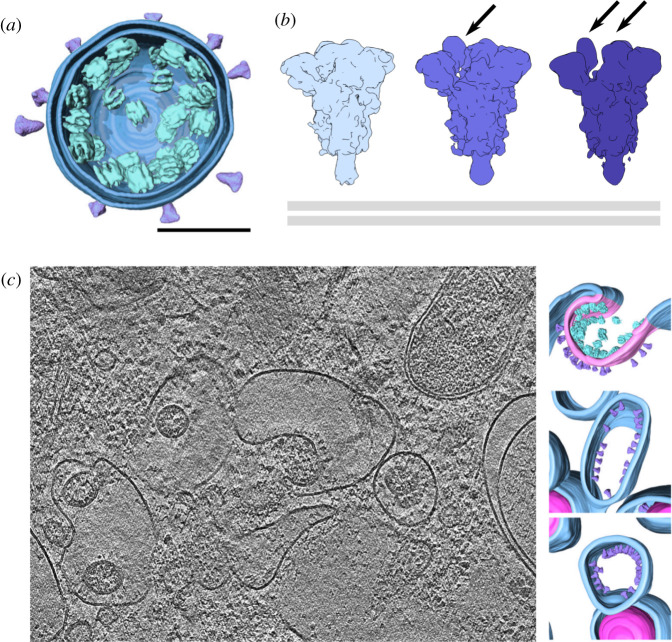
Cryo-electron tomography (cryo-ET) allows visualization *in situ.* (*a*) Three-dimensional volume rendering of a single virion derived from cryo-ET. The spike glycoprotein (purple) and viral ribonucleoprotein complexes (cyan) are subtomogram averages placed at their corresponding locations in the tomogram with vRNP orientations randomized. This panel is adapted from ref. [53] and licensed under CC BY 4.0. To view a copy of this license, visit https://creativecommons.org/licenses/by/4.0/. (*b*) Subtomogram averages of spike protein from the surface of virions. From left to right: closed conformation (EMD-11494), one RBD up (EMD-11495), two RBDs up (EMD-11496). RBDs in the up conformation are indicated by arrows. The position of the membrane relative to the glycoprotein is indicated by the double grey lines. (*c*) A tomographic slice showing SARS-CoV-2 budding events at ER–Golgi intermediate compartment membranes in VeroE6 cells (EMD-11863) with segmentation of different membrane events at right. The segmentation is adapted from ref. [53] and licensed under CC BY 4.0. To view this license, visit https://creativecommons.org/licenses/by/4.0/.

Leveraging the improved technologies described above, cryo-ET image reconstructions yielded exquisitely detailed structures of intact SARS-CoV-2 virions. Subtomogram averages of the spike trimers within these reconstructions were determined to subnanometre resolutions (figure 4*a*,*b*) [53–56]. Tomography combined with STA advantageously not only provides the protein structure but also the protein distribution, and the SARS-CoV-2 tomograms reveal the varying orientations and distributions of spike trimers on the virion surface, in addition to ultrastructural information on ribonucleoprotein assemblies within the virus [55]. Analysis of the tomograms showed that the spike trimers were more flexible than expected, with multiple hinge points resulting in various tilt angles relative to the lipid membrane [54,56]. Some trimers were also seen to adopt the postfusion conformation rather than the prefusion conformation, suggesting premature triggering of the metastable glycoprotein [54]. The presence of the postfusion trimer has clinical implications, not only because the prefusion state is needed for the virion to be infectious but also because different antigenic epitopes are exposed in the pre- and postfusion conformations.

Cryo-ET has also been used to image SARS-CoV-2 virions *in situ,* inside infected cells. Frozen vitrified cells were milled down to 200 nm-thick lamella using a focused ion beam (FIB) to generate a sample thin enough to be penetrated by an electron beam. Viruses were then imaged within the subcellular compartments associated with viral replication [53]. Individual viral RNAs with branched secondary structures were observed and viral proteins were found at sites with increased membrane curvature in the cryo-ET reconstructions, representing nascent virus particles captured at intermediate assembly steps (figure 4*c*) [53]. Hence, these types of cryo-ET visualizations have provided invaluable mechanistic insights on vital processes in the life cycle of SARS-CoV-2.

## 5. X-ray crystallography

**Figure 5.**
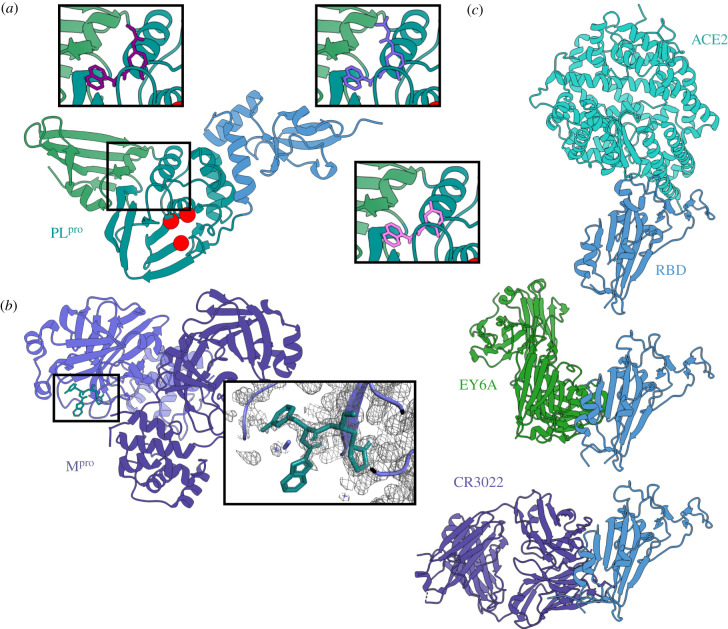
Crystal structures still provide the most detailed protein structures. (*a*) A cartoon representation of the crystal structure of the papain-like protease (PL^pro^) from nsp3 of SARS-CoV-2 (PDB 6WZU) with red spheres indicating the residues of the catalytic triad and the prototypic ‘thumb–palm–fingers’ architecture annotated in blue, teal and green. Three different inhibitors that bind to the same pocket are shown in the insets (PDB 7JIR, 7JIT, 7JIW). (*b*) The crystal structure of the SARS-CoV-2 M^pro^ (nsp5) homodimer bound to the 11b inhibitor in teal (PDB 6M0 K). The inset shows how density for the inhibitor can be observed in the crystallographic map. (*c*) Cartoon representations of the SARS-CoV-2 receptor binding domain (RBD; blue) bound to three different ligands. From top to bottom: ACE2 (sea green), EY6A Fab (green), CR3022 Fab (purple).

The proportion of cryo-EM structures being determined at resolutions sufficient to support drug design (approx. 3 Å) is increasing but remains a minority. Thus, most structure-guided drug discovery efforts continue to rely on crystallographic structure determination. Using crystal structures to help identify small molecules targeting SARS-CoV-2 proteins has been an active and productive area of research. Crystal structures of the proteases from HIV and HCV contributed to the development of antiviral protease inhibitors. In combination with some of the polymerase inhibitors mentioned above, protease inhibitors are essential components of current treatments against HIV and HCV. Crystal structures of the papain-like protease (PL^pro^) and the main protease M^pro^ (also known as 3CL^pro^) from SARS-CoV-2 have been determined, including some in complex with candidate inhibitors (figure 5*a*,*b*) [57–61]. These structures provide a powerful basis for the design of SARS-CoV-2 protease inhibitors. This structure-based design strategy remains one of our best hopes for developing an effective pharmaceutical treatment for COVID-19.

In addition to the protease structures, landmark structures of the spike receptor-binding domain (RBD) were determined by X-ray crystallography. The RBD is the region of the S protein that recognizes and binds the host cell, through the ACE2 (angiotensin-converting enzyme 2) receptor [62]. Many neutralizing antibodies halt infection by preventing receptor binding. Several of the SARS-CoV-2 variants of concern contain mutations in the RBD, most notably E484K, that increase ACE2 binding affinity or decrease antibody binding affinities (or both) [63,64]. The crystal structures of RBD bound to a soluble fragment of ACE2 are, therefore, some of the most important structures for understanding how SARS-CoV-2 recognizes host cells and how mutations in new virus isolates are likely affect this recognition (figure 5*c*) [65–67]. Several crystal structures of the RBD with various mutations bound to neutralizing antibody fragments are also available [30]. Together, the crystal structures of SARS-CoV-2 proteins paint a picture of the sites of vulnerability within the virus, and the extent to which they are conserved in different isolates or variants. This information can be used to develop therapeutics such as monoclonal antibodies or immunogens that are effective against a specific variant or set of variants.

## 6. Outlook

The spread of SARS-CoV-2, accelerated by global interconnectedness in our transport and trade links, has taken a calamitous toll and changed the way we live. However, the timing of the outbreak was fortuitous in that it followed technological breakthroughs in vaccinology and structural biology of a rare magnitude. These breakthroughs allowed us to visualize how SARS-CoV-2 interacts with cellular components in greater detail and at a faster pace than would have been possible even only a few years earlier. High-resolution imaging of SARS-CoV-2 has powerfully catalysed efforts to develop vaccines and therapeutics, giving structural studies a central role in enabling delivery of new COVID-19 vaccines and therapies with unprecedented speed. Here, we have reviewed the principal approaches that have been applied to visualize SARS-CoV-2, the structures they generated, and why they were important. Recent advances, particularly in cryo-EM and cryo-ET, have opened a new era in structural biology, making many previously intractable targets amenable to molecular-level visualization. The uses and imaging power of the techniques described here will continue to grow, yielding invaluable mechanistic insights on vital processes in the life cycle of SARS-CoV-2 and other viruses.

Another trend that can be expected going forward is an increase in the number of integrative multiscale imaging studies that combine different approaches to obtain a more complete picture of a biological process. Cryo-EM and cryo-ET imaging fill a gap in the resolution spectrum of imaging modalities. Cryo-ET allows imaging of intact biological assemblies *in situ*, cryo-EM allows more detailed visualized of macromolecule assemblies, and the resulting structures from both techniques are constantly improving in resolution due to ongoing developments in the field. Flanking these approaches, light microscopy allows us to explore the time dimension and probe subcellular localization of multiple components using fluorescent markers at lower resolution scales. A current limitation of light microscopy is that the timescales are still relatively short compared to the timescale of infection. While this means it is difficult to definitively link characteristics of a tracked virion to observations later in infection, improvements in both length and resolution of such imaging may someday make this possible. For applications requiring the highest resolution, such as structure-based drug design, X-ray crystallography remains the gold standard, but nuclear magnetic resonance (NMR), a powerful technique for identifying binding interfaces and measuring protein dynamics, also has the potential to make future contributions [68]. A beautiful example of multiscale imaging applied to SARS-CoV-2 is the study by Bartenschlager and colleagues [16] combining fluorescence microscopy, TEM, SEM and cryo-ET image reconstruction to visualize the ultrastructural changes in cellular structures induced by SARS-CoV-2 infection. The imaging work carried out so far on SARS-CoV-2 has already yielded an exquisitely detailed picture of the virus and its interactions with the host [69,70]. Further integrative imaging studies are still needed, however, in order to improve our understanding of the SARS-CoV-2 life cycle at the molecular level.

The scientific community's exceptional mobilization on SARS-CoV-2 research and the confluence of technological advances in multiple areas, particularly electron microscopy, have led to progress in visualization of SARS-CoV-2 at a pace that exceeded all expectations. Imaging studies of SARS-CoV-2 have informed vaccine design and helped identify new treatment strategies. The momentum gathered in this field must be sustained if we want to control this pandemic and treat its aftermath. At the same time, we need further method development in electron microscopy and other areas of structural biology to continue at a full pace so that we are even better prepared for future outbreaks. While the emergence of new viruses is inevitable, if we continue on this trajectory of technological progress and prioritized resourcing of virus research, we will be better positioned to deal with future epidemics.

## Data accessibility

This article has no additional data.
